# Mapping the dynamics of learning communities about Dutch healthy weight approaches: a causal loop diagram

**DOI:** 10.1186/s13690-024-01468-1

**Published:** 2024-12-20

**Authors:** Maud J. J. ter Bogt, Kirsten E. Bevelander, Esther A. H. Kramer, Merel M. van der Wal, Gerard R. M. Molleman, Maria van den Muijsenbergh, Gerdine A. J. Fransen

**Affiliations:** 1 Primary and Community Care, Radboud University Medical Centre, Nijmegen, The Netherlands; 2 AMPHI Academic Collaborative Centre, Nijmegen, The Netherlands; 3 Institute for Management Research, Radboud University, Nijmegen, The Netherlands; 4 Pharos, The Dutch Centre of Expertise on Health Disparities, Utrecht, The Netherlands

**Keywords:** Learning community, Community of practice, Healthy weight, Community approach, Causal loop diagram, Collaboration

## Abstract

**Background:**

Learning communities (LCs) are increasingly used among multidisciplinary public health challenges, such as local healthy weight approaches. LCs aim to stimulate learning, collaboration and actions. Previous research has provided insights into the underlying elements of multidisciplinary LCs, but little is known about the perceived causalities of these elements. Therefore, limited is known about what can be done to leverage LCs. This study aims to gain insights into the perceived dynamics of multidisciplinary LCs during the starting phase, including variables, and interconnectedness between variables.

**Methods:**

To elucidate LC dynamics, all members of two separate LCs participated in a qualitative interview about experiences, perceived learning, and actions during the first six months. Interviews were analyzed thematically. Subsequently, a qualitative causal loop diagram was designed.

**Results:**

The qualitative causal loop diagram showed three intertwined themes. The first theme explains why group dynamics are essential, and how jointly arranging the LC may optimize group dynamics. The second theme explains how insights are obtained through multidisciplinary knowledge exchange. The third theme explains how actions are executed when conditions are met. These LC group dynamics, learning and action influenced one another.

**Conclusions:**

To optimize LCs, it is highly recommended that stakeholders arrange them jointly, involve the appropriate partners, match with LC members’ needs, and motivate members to execute action. LC facilitators are recommended to use the causal loop diagram to identify their bottlenecks and how to intervene in those to optimize the LC.

**Supplementary Information:**

The online version contains supplementary material available at 10.1186/s13690-024-01468-1.

**Table:** 

Text box 1. Contributions to the literature
• Learning communities (LCs) are increasingly used among public health challenges, but there is limited evidence on the overall dynamics of their functioning, as previous studies only described LC variables in isolation.
• Our study advances previous research by explaining the perceived interrelatedness of LC variables and their (un)intended consequences, which ultimately determine the functioning of multidisciplinary LCs.
• Key intervention points for optimizing LC functioning include enhancing group dynamics within LCs, facilitating multidisciplinary knowledge exchange, and establishing conditions to execute LC actions.

## Introduction

In response to continuously increasing overweight and obesity rates [1, 2], the Dutch government set the goal to reduce overweight and obesity in the Netherlands from 50 to 38% by 2040 [3–5]. Therefore, many municipalities implemented healthy weight approaches (HWAs) in collaboration with multidisciplinary partners, for example, care professionals, municipality policymakers, and citizens [6, 7]. However, those HWAs had a limited effect on population weight reduction [8, 9], partly because the complexity of obesity was insufficiently recognized [10–12].

In 2021, learning communities (LCs) were established in five municipalities in the Gelderland region of the Netherlands to address this complexity and enhance HWAs’ impact. This approach was based on previous research suggesting that LCs are effective in achieving these goals [13, 14]. They chose to apply LCs, because collaboration between a multidisciplinary group of professionals is essential to achieve the joint objective of effective HWAs, and LCs aim to stimulate members to learn from one another, collaborate, and align their actions [15]. This is also why LCs are increasingly used among multidisciplinary public health challenges. We define LCs as “HWA stakeholders who get together during LC meetings to learn, collaborate and align or adjust actions to strengthen their HWA work”. Within LCs, LC members reflect on their professional practices in an exchanging, cooperative, inclusive, and continuous way that is focused on growth and learning [15–17].

LCs are rooted in organizational learning [18], of which Professional Learning Communities originate from education in schools [19–22], and Communities of Practice originate from informal networks [23–25]. The LCs consisted of elements of both Professional Learning Communities and Communities of Practice. In line with Communities of Practice, LCs include for example elements like flexibility, and are focused on sharing knowledge, building relationships and learning from other stakeholders with whom LC members do not work on a daily basis [23–25]. This corresponds to the importance of cross-domain collaboration within HWAs [9, 26, 27]. Moreover, in line with Professional Learning Communities, LC meetings had for example reflective dialogues, collective focus on learning, a common goal and supportive collaborative organizations [19–22].This corresponds to previous literature that suggests that reflecting and learning together is needed to strengthen HWAs [26, 28].

It is challenging to establish monodisciplinary LCs (i.e., LCs within one organization and/or with individuals performing relatively comparable work tasks/functions) that lead to both learning and action [15, 29]. Establishing multidisciplinary LCs (i.e., across multiple organizations and/or individuals performing diverse work tasks/functions) is even more challenging, due to the large differences in LC members’ daily tasks, working methods, and perceptions [15, 29]. Literature is scarce regarding LCs in multidisciplinary contexts, making it even more difficult to establish successful multidisciplinary LCs where LC members collaborate, learn and jointly come to action. Literature about monodisciplinary professional LCs in school settings has suggested important LC elements, including that LCs focus on processes to stimulate change and learning; context including group dynamics, size, location, phase, inclusive membership, mutual trust, openness regarding networks; and external influences including the community outside the LC and policy decisions [15, 30, 31]. For example, when members mutually trust one another, they may be more likely to openly share and reflect on their experiences, and consequently learn together. In addition to these elements found in school-based professional LCs, research in the social domain has identified other LC elements such as the broader community context (e.g., strategic vision), organization processes (e.g., transparency, clear responsibilities), creating synergy (e.g., short contact moments in between LC meetings, supportive backbone), making learned lessons explicit, and implementing results (e.g., finances, implementation strategies) [32]. More specifically, LC members explicitly mention what they have learned contributes to further learning. Subsequently, gained insights from the LC are more likely to be translated toward action, and more likely to be implemented [33, 34]. This illustrates not only the importance of clearly expressing the lessons learned, but also how it is connected to other elements. Although previous literature has provided lists of important elements for LCs, these elements are described in isolation. For example, group dynamics is described in terms such as team culture, loyalty and identification with the team, respecting individual differences, and balancing hierarchy and empowerment to create equal distributions within the team [15]; but the perceived causes of these group dynamics and the perceived effects of (in)adequate group dynamics are barely described.

Thus, previous research has identified key elements that are perceived as important in LCs, but it has not provided much insights into the perceived causalities among these elements, particularly in multidisciplinary LCs [35]. Consequently, there is limited knowledge about how to optimize LCs, while LC facilitators and members need more understanding into how LCs function to be able to further improve learning, collaboration and actions. To address this gap, it is crucial to gain better understanding of these perceived interrelatedness of causalities. This helps to explain potential (un)intended consequences while providing valuable insights into addressing experienced bottlenecks effectively. While some quantitative monitoring tools provide insights into the functioning of LCs (e.g., collaboration within LCs) [36–38], they focus only on pre-determined variables and fail to openly map how LCs are experienced. To address this limitation, we propose using a qualitative causal loop diagram (CLD). This is a visualized map that explains how LCs are experienced by means of words (in the form of variables) and directed arrows (perceived causal connections between these variables) [39]. The map helps to identify perceived (un)intended consequences, observed bottlenecks and possible solutions for successful multidisciplinary LCs. These insights can optimize LCs, enabling HWA stakeholders to learn, reflect, and adequately implement HWA actions [13–15, 19–25, 29]. This may result in better organized HWAs that aim to increase healthy weight prevalence on the long term. As it is likely that the LC starting phase sets the trend for future LC meetings, this study focused on the early phase of an LC. Therefore, this study aims to gain insight into the overall perceived dynamics of the functioning of multidisciplinary LCs during the starting phase, including the variables, and interconnectedness between variables.

## Methods

### Study design

This qualitative study investigated two LC groups in line with the consolidated criteria for reporting qualitative research (COREQ) checklist [40]. Semi-structured interviews were conducted about LC members’ experiences and expectations regarding LC participation, learning, and calls to action after the first two LC meetings. Based on the interview data, a qualitative causal loop diagram (CLD) was created. The need to obtain approval for the study was waived by the ethics committee of Radboud University and Medical Center (registration number 2021–13172). The ethical principles of the Declaration of Helsinki and GPDR regulations were followed. All participants received an information letter and gave written informed consent.

### Study setting

Both LCs consisted of a relatively stable group of HWA stakeholders (including citizens) from two to three adjacent municipalities. Each of these municipalities already had somewhat similar existing HWAs. Diverse HWA stakeholders were selected by health brokers from the municipal health service and/or municipality policy advisors, and sent a LC invitation letter. Citizens interested in strengthening existing HWAs were recruited via advertisement posters displayed in public spaces, as described elsewhere [41]. LC participation was voluntary; however citizens received a volunteer compensation, and a few HWA stakeholders from one LC received an allowance if they had no prior collaboration with the municipality. This resulted in 18 LC members in group A and 12 LC members in group B for LC meeting 2 (Table 1, description of every (job) function is described elsewhere [26]). Moreover, one steering group was formed across both LCs in which each municipality’s health councillor and external advisors from Dutch parties participated (e.g. The Dutch Centre of Expertise on Health Disparities, the Dutch association of municipalities, the Dutch healthy youth healthy future approach). A LC facilitator (the main researcher) was appointed (MB).

**Table 1:** Members’ function and presence – causal loop diagram about learning communities in five Dutch municipalities, 2022

(Job) function at LC meeting 2	LC group A (***n***)	LC group B (***n***)
Municipality policy advisors	3	2
Municipal health service’s health brokers	3	2
Care professionals (e.g., general practitioner)	4	2
Practice professionals (e.g., welfare worker)	4	4
Citizens	4	2
**Number of members at LC meetings**	**LC group A (** ***n*** **)**	**LC group B (** ***n*** **)**
Total members at LC meeting 1	16	12
Members present at LC meeting 1	13 (81.3%)	8 (66.7%)
Total members at LC meeting 2	18	12
Members present at LC meeting 2	17 (94.4%)	12 (100%)

The first LC meeting took four hours and focused on members getting to know one another and exchanging experiences with current HWAs. During the second four-hour LC meeting, members agreed that the LC would focus on direct causes of overweight (e.g., healthy nutrition) and underlying causes (e.g., poverty) among adults and children. The observe-reflect-plan-act cycle was applied via reflexive monitoring methods [42, 43] (described in detail in Additional file 1). As part of the observe phase, the research team studied the HWA (i.e., deployment of all activities, facilities, and policies; how the HWA was organized; leverage point themes to strengthen HWAs) in between LC meetings (Additional file 2); and members observed the HWA from their job-function perspective and observed the execution of actions originating from the LCs. These observations were reported during LC meetings (Additional file 2). Subsequently, members reflected and created LC plans relating to their work. Finally, actions were formulated in terms of learning questions on a dynamic learning agenda [42], and members initiated working groups per formulated action. Applying these actions into current work practices may contribute to improving the existing HWAs. The facilitator provided annual progress updates on the LCs to the steering group. Further, the LC was monitored through researchers’ observations during LC meetings, quantitative questionnaires, and qualitative interviews of members (including the current study). Subsequently, the LC facilitator reported these research findings during LC meetings, and LC meetings were adapted based on these findings as desired by members.

### Participants

All members were invited via e-mail for an online interview about the first two LC meetings. If potential participants did not respond, they received a reminder e-mail after two weeks and were phoned one week later. In total, 30 members (and two successors who had already been present during a LC meeting) participated in an interview of approximately 20 min. With a 100% response rate, this ensured a comprehensive capture of all LC experiences.

### Data collection

Based on prior monitoring tools, a semi-structured interview guide was designed to gather data about the LC starting phase [42, 44, 45]. The interview guide was pilot tested three times with health brokers from the municipal health service who worked in a different municipality. Although not involved in our LCs, these broker had experience in similar collaborations. Based on their feedback, small adjustments were made to the interview guide, such as changing specific words to create less wordy sentences. Members were asked open questions about their experiences regarding LC evaluation, LC goals and roles, learning from monitoring, acting after the LC, individual learning in the LC, and learning interaction with broader network (Table 2; Additional file 3) [42, 44, 45]. Interviews were conducted in Dutch by an independent and trained researcher (LT, MSc intern, female), and members had the opportunity to introduce their own topics. Moreover, after the second LC meeting, members filled in an evaluation questionnaire that was constructed through literature and input from LC members and experts, as further elaborated elsewhere [46]. For the current study, only open-ended questions were analyzed. These questions evaluated LC meetings with regard to positive aspects, areas for improvement, members’ LC meeting output, members’ next actions, and how this LC output and actions contributed to members’ HWA goal.

**Table 2:** Interview topics and questions – causal loop diagram about learning communities in five Dutch municipalities, 2022 [42, 44, 45]

Topic	Sample interview questions
LC evaluation	Think about when you were in the LC meeting. What thoughts did you have? What did you like? What did you not like?
LC goals and roles	What do you think the LC is working toward? What needs to happen to get there?
Learning from monitoring	How do you experience adapting your work activities in the LC?
Acting after LC	How does the learning community influence what you do?
Individual learning in LC	What have you gained from the LC so far?
Learning interaction with broader network	How have you involved your partners who are not LC members?

### Data analysis

Data analysis consisted of two steps.

#### Step 1: thematic analysis

Interviews were voice-recorded and transcribed ad verbatim. A thematic analysis was performed per LC using Atlas.ti version 9 software from the perspective: (1) How are the LC meetings perceived by the LC members? (2) What do LC members learn about HWA after participating in the LC? (3) What underlying mechanisms play a role for LC members to execute actions after LC participation? [47]. First, two coders (MB, EK) jointly applied open coding to one transcript while discussing the codes. Second, seven interview transcripts were coded by both coders individually, followed by a discussion on differences and similarities until consensus was reached (MB, EK). This resulted in an inductive conceptual coding structure that was discussed by the two coders and one of the co-authors (MB, EK, KB), after which small adjustments were made to the coding structure. For example, codes about learning were applied when the transcript contained words such as idea or insight. Third, identical codes were merged by both coders (MB, EK), resulting in a merged coding structure. The merged coding structure was then consistently applied by one coder until all interviews were coded (EK) and by another independent coder until the qualitative answers in the evaluation questionnaire were coded; new codes and ambiguous segments were discussed with the second coder (MB). Fourth, the coded data were discussed by three authors per LC group, and codes were grouped into subthemes (MB, EK, KB), and afterwards discussed, ordered, and categorized (MB, EK, KB) (Additional file 4).

#### Step 2: causal loop diagram

Based on the qualitative interview data, a qualitative CLD was created. This is a conceptual model that illustrates how LCs are experienced by LC members. The model shows variables (subthemes from step 1) that are linked through perceived causal connections [39]. Every connection has a polarity, where a positive polarity means that the direction of change stays the same (e.g., an perceived increased cause was perceived to led to an increased perceived effect), and a negative polarity means that the direction of change becomes the opposite (e.g., an perceived increased cause was perceived to led to a decreased perceived effect). These causal connections may eventually reinforce or balance its initial variable (also called feedback loop), which subsequently displays how solutions are strengthened or problems are weakened.

A CLD to map the dynamics of multidisciplinary LCs was created during an iterative approach that applied four steps based on existing methods [48]. First, the main author (MB) marked all themes and subthemes in the coding structures of both LCs as a variable. As all variables and connections of both LCs overlapped, one CLD for both LCs was created. Second, the interpretation of the perceived negative or positive causal connections (i.e., polarity) between these variables were checked based on LC observations, and substantiated with implicit and explicit quotes from interview transcripts by going through all transcripts (MB). Throughout this process, variables were split up or combined when the content of variables and their connections overlapped [49]. For example, *Insights into points of improvement HWA*, *Insights into HWA are complex*, and *Knowledge about HWA initiatives* were combined into *Better understanding HWA complexity*. Afterwards, interview transcripts were read thoroughly to identify potentially missed perceived causal relationships until among three subsequent interview transcripts no new relationships were found (MB). Third, the variables and perceived connections were drawn in Vensim PLE 10.1.0 to create the first version of the CLD. Fourth, the CLD was discussed and themes were identified until consensus was reached with the other coder (MB, EK), two other co-authors (MB, KB, GF), and a qualitative systems modelling expert (MB, MW). Small iterations were made, such as excluding direct connections that were indirectly connected through another variable. Lastly, as various feedback loops largely overlapped, the unique feedback loops were identified (MB), as written in the results section. The CLD and results were translated to English, and drawn in the software program Kumu.

## Results

**Fig. 1:**
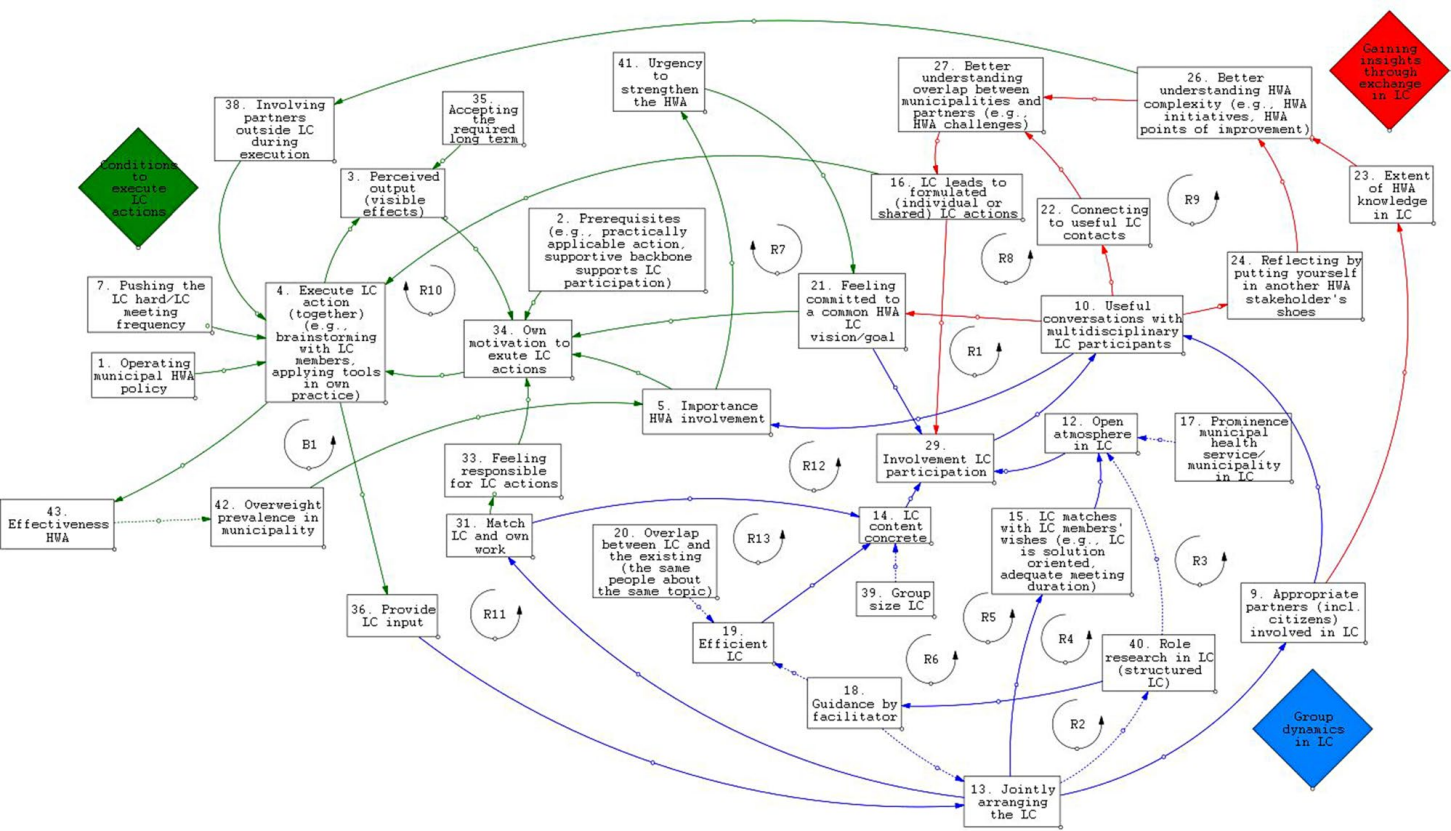
Causal loop diagram about Learning Communities’ starting phase in five Dutch municipalities, 2022

These results are based on two LC groups, but the CLD dynamics were coherent in both groups. Figure 1 (more readable in Kumu [50]) shows that the CLD consisted of 50 perceived connections between 35 variables describing both behaviors (e.g., *provide LC input*) and perceptions (e.g., *open atmosphere in LC*) (Additional file 5). Most importantly, 13 reinforcing feedback loops were found to strengthen initial LC experiences, and one balancing loop was found to counteract initial LC experiences.^1^ The CLD included three themes, indicated by the colored diamonds: “Group dynamics in LC”, “Gaining insights through exchange in LC”, and “Conditions to execute LC actions”.

*Arrows with a solid line indicate positive polarity*,* meaning that the direction of change stays the same. In other words*,* when a cause was perceived to increase (was perceived to decrease)*,* the effect also was perceived to increase (was perceived to decrease). Arrows with a dotted line indicate negative polarity*,* meaning that the direction of change becomes the opposite. In other words*,* when a cause was perceived to increase (was perceived to decrease)*,* the effect was perceived to decrease (was perceived to increase). The color of the arrow indicates the theme to which it belongs. Variable names of the CLD appear in the written text in italic. See Kumu for the more readable CLD* [50].

### Group dynamics in LC

Several feedback loops within the theme “Group dynamics in LC”, partially overlapped and covered mostly different ways in which the LC may match with members’ desires, such as the variables *research role in LC*, *right partners participate in the LC*, *LC is concrete*, and *open atmosphere in LC*. The CLD revealed the importance of a democratic approach to decision making in the LC by more *jointly arranging the LC*, which was perceived to relate to *members’ LC participation involvement* and *feeling committed to a common HWA LC goal*, and consisted of three pathways.

First, when the *LC was arranged jointly* (i.e., the LC members and facilitator together) to a bigger extent, it was believed to result in more *involved appropriate partners in the LC*, which was perceived to result in more *useful conversations with multidisciplinary members*. This was perceived to result in both an increased *importance of members’ HWA involvement* and an increased *feeling of commitment to a common HWA LC goal*, the latter of which is illustrated by the following quote:

*And of course we started with healthy weight. We have opened it up a bit by agreeing that we should not focus only on healthy weight*,* so I think we want to achieve the same goal together. And that is best for the citizens. So that’s going well and I think we are with a nice group of people.*

Both an increased *importance of members’ HWA involvement* and an increased *feeling of commitment to a common HWA LC goal* was perceived to increase members’ *motivation to execute actions*, leading to more *executed LC actions*. This increased the *LC input members provided* and subsequently was perceived to led to a *LC that was jointly arranged* to a higher degree. All polarities described in the results section also apply the other way around, in this example meaning that when less *LC input was provided*, this was perceived to led to a *LC that was jointly arranged* to a lesser extent.

Second, the more the *LC was jointly arranged*, the more it was perceived that the *LC matched with members’ wishes*. A better *match between the LC and members wishes* resulted in a more *open atmosphere within the LC*, thereby increasing members’ *LC participation involvement.* Therefore, more *useful conversations with members* took place, increasing members’ *feeling of commitment to a common HWA LC goal* and consequently increasing *members’ LC participation involvement*. More *useful conversations with multidisciplinary members* was also perceived to increase the *importance of HWA involvement*, increasing *members’ urgency to strengthen the HWA*. Perceiving *HWA involvement as more important*, and feeling more *commitment to a common HWA LC goal* both were perceived to led to more *motivation to execute LC actions*, as illustrated by the following quote:

*It helps. Yes*,* I mean*,* now I sit here again… I sit with like-minded people*,* so everyone also thinks it’s important that you are busy with lifestyle. … Otherwise*,* I’m a loner. And it feels like a kind of warm bath to be with people who also think: yes*,* I believe there is still a lot to be gained here.*

This was perceived to led to more *executed LC actions*, then to providing more *LC input*, and consequently to more *joint arrangement of the LC*.

Third, *jointly arranging the LC* was also perceived to influence the *role of research in the LC* and the *guidance provided by the facilitator*. More specifically, if the *LC was arranged with LC memb*ers to a higher degree, this was perceived to led to a smaller *role for research in the LC* (where research refers to a structured LC, e.g., using stickers to vote and set priorities). On the one hand, this resulted in a more *open atmosphere in the LC*, which subsequently was perceived to increase *members’ LC participation involvement*. On the other hand, a smaller *research role in the LC* resulted in less *guidance provided by the facilitator*, which increased the extent to which the *LC was jointly arranged*. Simultaneously, the less *guidance the facilitator provided*, the less the *LC was perceived as efficient*. This was perceived to increase the extent to which the *LC was concrete* (e.g., members had more possibilities to share examples and opinions), thereby increasing members’ *LC participation involvement*. This polarity also appeared the other way around, meaning that, when the *LC was less concrete*, this was perceived to decrease *members’ LC participation involvement*, as illustrated by the following quote: “*I just get itchy from talking too much and not making things concrete enough*,* so yes*,* I find that difficult*,* so to speak.*” The increased members’ *LC participation involvement* then was perceived to led to *jointly arranging the LC* more via the same variables as described in the second pathway (in the previous paragraph).

Lastly, although *prominence of the municipality in the LC* was not part of a feedback loop, it was perceived to inhibit the *open atmosphere*. This polarity also appeared the other way around, meaning that, when the *municipality was less prominent in the LC*, this was perceived to increase the *open atmosphere*. Another variable that was not part of a feedback loop was *group size*. *Group size* inhibited *concreteness of the LC*, meaning that, if the *group size* was bigger, the *LC was perceived as less concrete*.

### Gaining insights through exchange in LC

The theme “Gaining insights through exchange in LC” emphasized the importance of high LC participation and useful conversations during LCs. The theme showed that when members felt more *involved in LC participation*, this was perceived to led to more *useful conversations with multidisciplinary LC participants* during LC meetings. On the one hand, more *useful conversations* were perceived to lead to feeling more *connected to useful LC contacts*, which resulted in better *understanding how HWAs overlapped between municipalities and partners*. On the other hand, when more members perceived their *conversations as useful*, this was perceived to led to more *reflecting*. This was perceived to lead to better *understanding of HWA complexity* (e.g., insights into HWA initiatives, HWA points of improvement), which also led to a better understan*ding of how HWAs overlapped between municipalities and HWA partners*. These insights were perceived to lead to more *formulated individual or shared LC actions*, which further increased *LC participation involvement*.

### Conditions to execute LC actions

LC actions included for instance brainstorming with members and applying tools in members’ own practice. The “Conditions to execute LC actions” theme described three pathways related to *motivation to execute LC actions*. First, the more *LC actions were executed* by members, the more they *perceived LC output*. This increased their *motivation to execute LC actions*, which then was perceived to lead to more *executed LC actions*.

Second, fewer *executed LC actions* was perceived to lead to the *HWA being less effective*, which was viewed as increasing *overweight prevalence* in the long term. This perception increased members’ *perceived importance of HWA involvement*, which then was perceived to increase their *motivation to execute LC actions* and therefore increased the *executed LC actions*, as illustrated by the following quote: “*Then we are asked to participate*,* and we do so because we think it is important*,” which thus balances the initial variable. This pathway illustrates that less *executed LC actions* were perceived to eventually stimulate more *executed LC actions*.

Third, the more members *executed LC actions*, the more they perceived to *provide LC input*, as illustrated by the following quote regarding an action to formulate a municipality-wide vision:

*Well*,* with one another that shared vision or that shared framework [i.e.*,* our shared LC action]*,* next Monday we happen to get together again. … Actually formulating that together*,* setting it up*,* and taking it to the learning community next time and discuss it there*,* this is our view*.

Members *providing more LC input* was perceived to result in a *LC that was jointly arranged* to a higher degree, which was believed to lead to a better *match between the LC and members’ own work*. This perception increased the extent to which me*mbers felt responsible for LC actions*, thereby again increasing the extent to which members *executed LC actions*. Simultaneously, *feeling responsible for LC actions* also indirectly increased the *LC actions executed* by members through members being more *involved in LC participation*, resulting in more *useful conversations with multidisciplinary LC participants* and therefore was perceived to increase the *importance of HWA involvement*.

Lastly, several variables that were not part of a feedback loop were also perceived to influence the extent to which members executed LC actions. For example, some municipalities had a more *operational municipal HWA policy* (e.g., health was covered to a greater extent in municipal policies), which was believed to increase the extent to *which LC actions were executed* by members. This positive polarity between these variables also indicated that municipalities with less *operational municipal HWA policy* were perceived to inhibit the extent to which *LC actions were executed*, as indicated by the following quote:

*Well*,* partly also the confirmation that there really needs to be a foundation before anything else can be built. This confirms once again that we still have to get a lot of things in order in [municipality name]*.

### Interconnected themes

The themes were interconnected, which elucidates the importance of members setting the LC agenda and involving external HWA partners in LC action execution. More specifically, when members *executed more LC actions*, members also perceived to provide more *LC input*, which then was perceived to increase the extent to which the *LC was jointly arranged*. This was perceived to cause more *appropriate partners to become involved in the LC*, resulting in two pathways. First, more *appropriate partners in the LC* was perceived to lead to more *useful conversations with multidisciplinary LC participants*, which then led members to feel *better connected to one another*. This was perceived to result in a better *understanding of how HWAs overlapped between municipalities* and then led to the *formulation of more LC actions*. Second, more *appropriate partner involvement in the LC* was also perceived to increase the *HWA knowledge in the LC*, as illustrated by the following quote:

*I think what I have gotten the most out of it [the learning community] so far actually is the input from those partners. What comes to their mind among certain questions and what do they think about certain things? And then I don’t really mean [names]*,* because I actually do know it from them. But then especially those that are a little further away or the citizens who thought along*.

This greater *HWA knowledge in the LC* was also perceived to increase the *understanding of HWA complexity*. Therefore, members *involved more partners outside the LC* during LC action execution. Both pathways were believed to further increase *LC action execution*, as also illustrated by the following quote:

*You have then done a kind of quite extensive inventory and exploration together and perhaps put together some solutions. Then you really have to professionalize at that moment and really go involving parties and implementation in order to actually realize this and ensure that concrete actions are being taken in that municipality*.

## Discussion

To our knowledge, this is the first study to use a CLD to gain insight into the overall dynamics of the functioning of multidisciplinary public health LCs during the starting phase, including the variables, and interconnectedness between variables. The CLD is a conceptual model that can deepen the understanding of LC functioning. The CLD described three themes. The first theme explains why group dynamics (e.g., *open atmosphere*) are essential for functional LCs (e.g., *LC content concrete*) and how group dynamics may be optimized by jointly arranging the LC (e.g., *provide LC input*,* LC matches with LC members’ wishes*). The second theme explains how learning (e.g., a *better understanding of HWA complexity*) happens through multidisciplinary knowledge exchange (e.g., *useful conversations*,* reflection*). The third theme explains how formulated actions may be performed when certain conditions are met (e.g., *personal motivation to execute LC actions*). All themes were intertwined, meaning that LC group dynamics, learning and action influence one another. This also emphasizes the importance of members setting the LC agenda and involving external HWA partners in LC action execution.

In line with previous studies, our study has indicated group dynamics, learning, and action as important to LC functioning and identified similar variables, such as LC context (e.g., group dynamics, size, openness regarding networks) and external influences (e.g., policy decisions) [15]. Nevertheless, our study has advanced previous research by explaining the interrelatedness of LC variables and their (un)intended consequences that determine the functioning of multidisciplinary LCs; these may be used to identify LCs’ bottlenecks and how to intervene in them to optimize LCs. The theme regarding group dynamics was previously described as an important individual variable for team culture and respecting individual differences [15]. However, previous studies did not explain the causes of (in)adequate group dynamics and the effects of (in)adequate group dynamics, whereas our CLD illustrates exactly that. For example, the CLD suggests that an *open atmosphere* originated directly from having a *LC that matches with LC members’ wishes*, a limited *research role*, and a limited *prominent role for the municipality*. Regarding these variables, one study identified shared repertoire as an important variable [51] related to having a *LC that matches with LC members’ wishes*; and another study identified tensions between roles as an important variable [52] related to a limited *prominent role for the municipality*. Notably, these studies also identified variables such as group composition, mutual engagement, reflective dialogues, and ownership, which all appeared as **indirect** causes and effects in our CLD [51, 52], meaning that this CLD achieved an advanced understanding of LCs compared with only describing the variables in isolation. As this illustrates that qualitative CLDs achieve further understanding of the interconnectedness of important variables and their (un)intended consequences within a theme, we recommend other researchers also to translate their thematic analysis into a CLD of their system of interest, especially when qualitative data targets a complex problem; includes open, elaborative stories; and focuses on the interrelatedness of root causes (i.e., interview protocol from a systems perspective) [48].

The CLD suggests important intervention points (i.e., leverage points), which are small LC changes that can lead to substantial changes into how the LC functions. These points thus affect specific variables and therefore feedback loops in a way that it optimizes LC functioning. First, the CLD indicates that a LC that is perceived as more *multidisciplinary* and less *dominated by municipal members* results in better *learning to understand HWA complexity*. The importance of the multidisciplinary approach was also reported in a previous study on student education [53]. Second, the CLD illustrates that a *larger LC group* results in LC meetings that are less *practically applicable* for all members. Therefore, LCs that are less multidisciplinary need to intervene to learn more understanding of HWA complexity (e.g., acquire input from HWA stakeholders outside the LC), whereas larger LC multidisciplinary groups need to intervene to make LC meetings more practically applicable for all members (e.g., working in subgroups during LCs). Third, the CLD suggests that LCs should be *jointly organized*, meaning that *members provide LC input* and the LC facilitator enables co-creation, regarding both the LC meetings and research conducted about the HWA. Previous participatory action research also indicates the importance of co-creation for LCs [54]. Altogether, LC facilitators and members are recommended to arrange the main feedback loops within their LC: *arrange the LCs jointly with members*, *involve the appropriate partners*, and *match the LC with members’ needs*, as this results in better *HWA insights* and therefore *formulated actions*. This recommendation was in line with previous research [32, 51, 52], but our CLD further explained the dynamics. Fourth, *members’ motivation to execute formulated actions* may be stimulated by their *perceived LC output*, *HWA involvement importance*, and *feeling responsible for LC actions* – all aspects mostly not reported in previous research [32, 51, 52]. Fifth, several exogenous variables, i.e., variables that are not part of a feedback loop, might be used to boost LCs, as they influence feedback loops but are not dependent on any other variables and – from this CLD – appear to be effective intervention points [55], such as *pushing LC meeting frequency*, *accepting the required long term* for LC output, *prerequisites* to perform LC actions, and *LC group size*. It is, however, likely – but has to be further researched – that these variables have an optimum, suggesting that both infinitely high and infinitely low *group sizes* are undesirable [56, 57]. Lastly, the CLD suggests that LCs might *strengthen HWAs* and therefore decrease *overweight prevalence* in the long term in the municipalities. LC members expect interventions to achieve these successful results, meaning that more research is needed regarding the impact of LCs on HWAs [35].

The CLD provides an overview of many interconnected variables and their possible (un)intended consequences that determine the functioning of LCs, and this may help to strengthen LCs in practice [35, 58, 59]. To achieve this potential, LC facilitators working on HWAs are recommended to critically review our CLD for their context by indicating the dynamics that also apply to their LC, as this may enable facilitators to better understand the functioning of the starting phase of their LC. Subsequently, they can use our or their own adjusted CLD as a scan to identify potential leverage points in their LC’s starting phase together with members by identifying the variables or themes that currently score as most insufficient (i.e., may be improved the most).

The actual bottlenecks and therefore essential intervention points may differ between LCs, meaning that how a LC may be improved may differ between LCs, as also suggested previously [52]. For example, in one LC, an *open atmosphere* may be a bottleneck, whereas this might be adequate in another LC. The polarities in the CLD illustrate the nature of the connections, but do not indicate whether the variable values are high or low, good or bad. Yet, understanding the value of each variable and connection could show their relative importance, helping to identify when certain variable values reach a threshold and become bottlenecks. This insight could guide LC facilitators and members in implementing interventions to address these bottlenecks. Therefore, future research is recommended to elucidate the relative importance of each variable and connection using stock and flow models.

### Strengths and limitations

The main study strengths include the interviews having a maximum response rate (data saturation) and being executed by an independent researcher. Connections and polarities in the CLD were substantiated with quotes from interview transcripts. As the CLD indicated that less focus on research in the LC would be beneficial for the LC, it was decided not to execute a member check about the CLD. Future research might perform a member check to add even more certainty about the validity of the CLD. Furthermore, the LC facilitator was highly involved in LC meetings and designing the CLD (MB). This dual role is a strength, as it facilitated the interpretation of the data in line with the LC context. However, the dual role might unconsciously have resulted in a bias toward certain interpretations. To prevent the latter, other researchers who were not involved in facilitating the LCs were highly involved throughout the data-analysis steps. Moreover, the CLD indicates interrelatedness of variables as perceived by LC members, which may not necessarily represent their actual interrelatedness. Further, the first six months of the LCs were studied, meaning that the LCs were still in development and members did not yet have a wide range of LC experiences and expectations. Previous research indicated that stakeholder network dynamics change over project phases [60]. This may explain why there were limited balancing feedback loops discovered. Therefore, these LC meetings will be monitored in future research to update the CLD after two years and identify LC dynamics over a longer term. Lastly, even though the CLD was designed on the basis of two LC cases, it might be likely that comparable dynamics apply to other LCs, as comparable variables were previously found in other LCs [15, 32, 51, 52, 54]. Still, caution is warranted when generalizing the current CLD for the starting phase of LCs. For example, LCs organized differently - such as being national rather than regional, or monodisciplinary rather than multidisciplinary - may not identify with all aspects of the CLD (e.g., specific words). Therefore, future research is recommended to make this CLD generalizable to all LCs about public health topics, and study LC dynamics in later LC phases as well.

## Conclusion

Our study is the first to present a conceptual model for LCs about HWAs. Our CLD demonstrates how three themes are perceived to influence the functioning of the LC starting phase. The first theme explains why group dynamics are essential, and how it may be optimized by *LC members jointly arranging the LC*. The second theme explains how insights are gained through multidisciplinary knowledge exchange. The third theme explains how formulated actions may be performed when certain conditions are met. All themes are intertwined, emphasizing the importance of *members setting the LC agenda* and involving external HWA partners in LC action execution. To optimize LCs, it is essential that *LCs are jointly arranged*, the *appropriate partners are involved*, and members are *motivated to execute actions*. LC facilitators are recommended to use a CLD to identify their bottlenecks and how to intervene in those to optimize LCs. Moreover, researchers are recommended to translate their thematic analysis into a CLD to gain a better understanding of their system of interest.

## Electronic supplementary material

Below is the link to the electronic supplementary material.

Supplementary Material 1

Supplementary Material 2

Supplementary Material 3

Supplementary Material 4

Supplementary Material 5

## Data Availability

The data that support the findings of this study are available from Radboudumc but restrictions apply to the availability of these data, which were used under license for the current study and so are not publicly available. Data are, however, available from the authors upon reasonable request and with permission of Radboudumc. Therefore, data may be requested by emailing the quality team of our department of primary care (kwaliteitsteam.elg@radboudumc.nl).

## Footnotes

1 A reinforcing loop ensures that the original variable score will become more extreme (i.e., if the variable decreases, the feedback loop will ensure that the variable ultimately decreases further; if the variable increases, the feedback loop will ensure that the variable will become even higher). A balancing loop does the opposite (i.e., if the variable reduces, the feedback loop will ensure that the variable increases again; if the variable increases, the feedback loop will ensure that the variable reduces again).

## Abbreviations

CLD: Causal loop diagram
HWA: Healthy weight approach
LC: Learning community
