# Supplementary material for: Mapping the dynamics of learning communities about Dutch healthy weight approaches: a causal loop diagram
Source: Arch Public Health. 2024 Dec 20;82:238. doi: 10.1186/s13690-024-01468-1 (PMC11660615; doi:10.1186/s13690-024-01468-1)
Supplement: Supplementary file 1 — Supplementary Material 1 [file 13690_2024_1468_MOESM1_ESM.docx]

**Additional file 1: Overview of LC meeting 1 and 2 content – causal loop diagram about learning communities in five Dutch municipalities, 2022**

| **LC** | **n  A \| B** | **Research input (observe)** | **Techniques used (methods in LC)** |
| --- | --- | --- | --- |
| **1 (October 2021)** | 15 \| 10 | Facilitator: none, only project presentation | - Get to know one another - Discussed cooperation within LC, i.e. roles and expectations by means of post-its and group discussion (Wagemakers et al., 2010). - [Wall of Wonder](https://instituutvoorinterventiekunde.nl/de-wall-of-wonder-een-instrument-dat-verbindt-door-barbara-van-kesteren/): mapped the content of the HWA per municipality in the past until the future, including previous attempts to strengthen the HWA, successes and failures - Plenary discussion about 1) differences and similarities between municipalities and 2) who or what is needed to strengthen the HWA - Practical agreements about LC |
| **2 (January 2022)** | 17 \| 13 | Facilitator: Results monitoring instrument to measure the strength of the health promotion system 2021 | [Online session]   - Plenary discussion about topics LC 1 (mutual goal, definition HWA) - Presentation results monitoring instrument to measure the strength of the health promotion system 2021 - Reflection on research input - Causal analysis on results monitoring instrument to measure the strength of the health promotion system (van Mierlo et al., 2010). - Actor analysis of HWA (van Mierlo et al., 2010). - Formulating actions during a plenary discussion, prioritizing them, and creating working groups per action. - Listing the actions on a dynamic learning agenda. (van Mierlo et al., 2010) |

*HWA = healthy weight approach; LC = professional learning community; n = number of LC members; A = LC group A; B = LC group B*

**References**

Van Mierlo, B.C., Regeer, B., Amstel, M., Arkesteijn, M.C.M., Beekman, V., Bunders, J., et al. (2010). Reflexive Monitoring in action. A guide for monitoring system innovation projects. Communication and Innovation Studies, WUR; Athena Institute, VU.

Wagemakers, A., Koelen, M.A., Lezwijn, J., Vaandrager, L. (2010). Coordinated action checklist: a tool for partnerships to facilitate and evaluate community health promotion. *Global Health Promotion*,17(3):17-28 . <https://doi.org/10.1177/1757975910375166>
