# Supplementary material for: Mapping the dynamics of learning communities about Dutch healthy weight approaches: a causal loop diagram
Source: Arch Public Health. 2024 Dec 20;82:238. doi: 10.1186/s13690-024-01468-1 (PMC11660615; doi:10.1186/s13690-024-01468-1)
Supplement: Supplementary file 2 — Supplementary Material 2 [file 13690_2024_1468_MOESM2_ESM.docx]

**Additional file 2: Gaining insights into current HWAs – causal loop diagram about learning communities in five Dutch municipalities, 2022**

**Methods**

The research team designed a logic model at the start of the project (MB, GF, KB, GM, MM). It was expected that when professional learning communities (LCs) were organized in line with certain conditions and inputs, output resulted in terms of learning and acting among members on the short term. Subsequently, medium long term outcomes were expected regarding strengthened organization of the healthy weight approach (HWA) (e.g., collaboration structure), and HWA content (e.g., activities and policies). On the long term, expected outcomes could include changes among citizens’ (e.g., HWA experiences, behavior, body mass index-z scores).


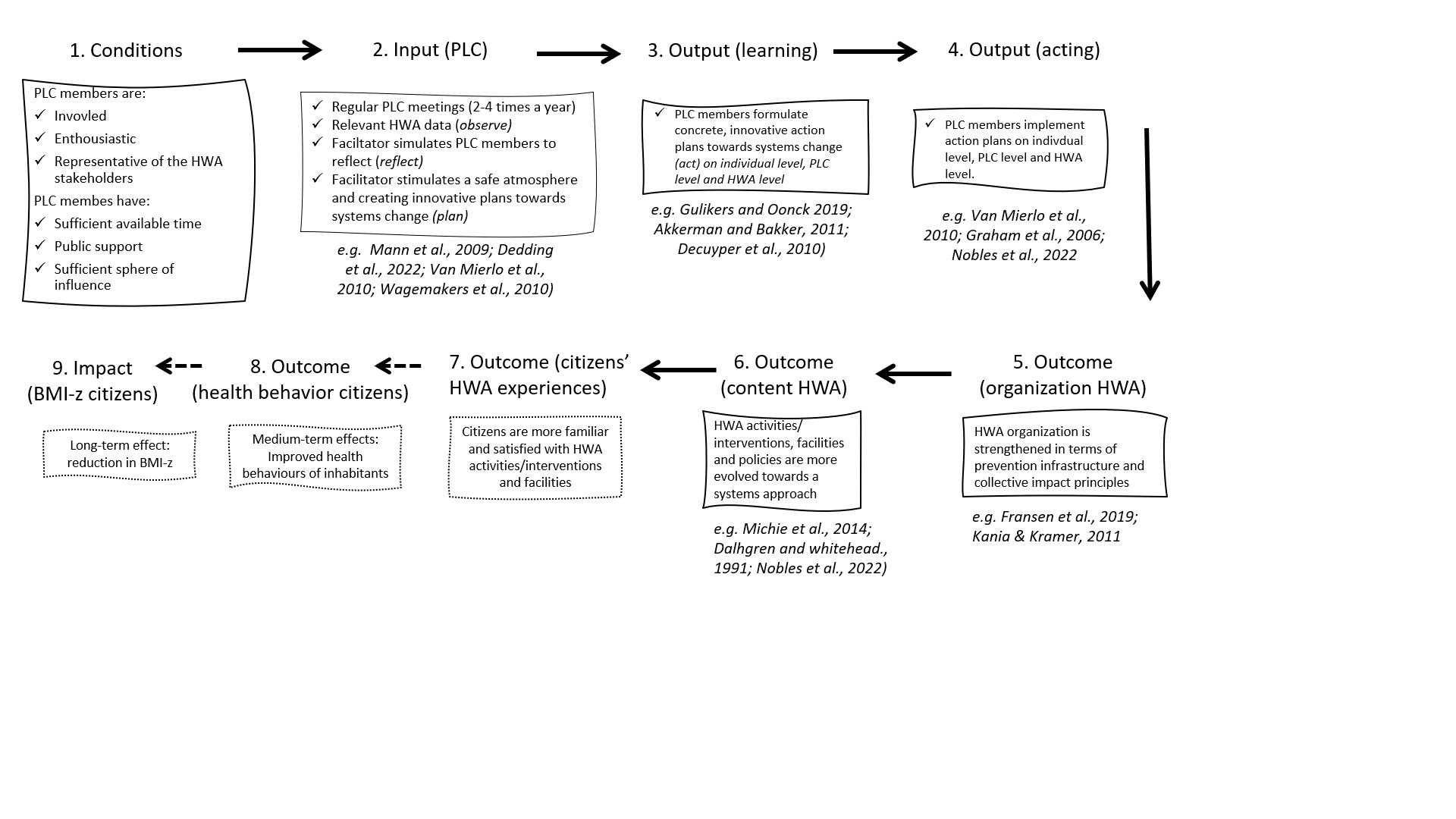


Based on this logic model, the research team decided what was needed to gain insights into current HWAs. During the first LC meetings, a focus group was organized where all HWA elements were mapped per municipality. Subsequently, these were scored on the Behavior Change Wheel (Michie et al., 2011), meaning that among all HWA elements its target group, target behaviour, source of behaviour, intervention functions, and policy categories were determined, based on previously developed methods (Dijkstra et al., submitted). Further, both citizens living in low SEP neighbourhoods (ter Bogt et al., submitted), and LC members were interviewed about the current HWA (ter Bogt et al., 2023); as described elsewhere. Lastly, the monitoring instrument to measure the strength of the health promotion system was conducted among members and their stakeholders who agreed to receive the questionnaire, to gain insights into the organization of the HWA (Fransen et al., 2019). This monitoring instrument is an online questionnaire that gains insights into collaboration, support, integrated approach, health monitor data assessment, visibility of results, reach of target population, sustainability and consistency of the HWA.

**Results**

In almost every LC meeting from meeting 2 onwards, a manageable amount of the results of a specific topic were reported back to the members. For example, a short presentation, video or infographic of a part of the results as described below was given.

With regard to the HWA elements, the focus group during LC meeting 1 indicated that all municipalities had 11 to 19 HWA activities (such as interventions), 1 to 3 approaches (such as Healthy Youth, Healthy future; Healthy school), 1 to 3 local agreements (such as prevention agreement), and 1 to 4 working groups.

Moreover, leverage point themes from a professionals (ter Bogt et al., 2023), and low socioeconomic position citizen perspective were derived, as described elsewhere (ter Bogt et al., submitted).

Besides, HWA elements scored on the Behavior Change Wheel indicated that the HWA elements mainly focused on the themes sports and movement, and nutrition; and had elements for all age groups. With regard to the sources of behaviour (Capability Opportunity and Motivation – Behavior model) HWA elements focused on physical capability, psychological capability, social opportunity, physical opportunity, and reflective motivation; but barely on automatic motivation. Further, with regard to intervention functions, HWA elements covered education, persuasion, training, environmental restructuring and enablement; but barely covered incentivisation, restrictions, modelling and coercion. Lastly, with regard to policy categories guidelines, service provision, environmental/social planning, communication/marketing were covered, but fiscal measures, regulation, and legislation were barely covered.

With regard to the HWA organization structure, the monitoring instrument to measure the strength of the health promotion system indicated per municipality which aspects were scored relatively high, and which elements were scored relatively low (e.g., reach of target population).

**References**

Dijkstra, S.C., van der Heijden, E., Klarenbeek, T., Ter Bogt, M.J.J., Renders, C., Verhoeff, A., van Nassau, F. (Submitted) Evaluation of evidence-informed working and systems thinking applied in a local community approach to prevent childhood overweight and obesity.

Fransen, G., Wagemakers, A., Molleman, G. (2019). The development of a monitoring instrument to measure the strength of health promoting systems. *European Journal of Public Health*, 29. 10.1093/eurpub/ckz185.611

Michie, S., van Stralen, M.M., West, R. (2011). The behaviour change wheel: A new method for characterising and designing behaviour change interventions. *Implementation Sci*, 6, 42 <https://doi.org/10.1186/1748-5908-6-42>

Ter Bogt, M.J.J., te Riele, Y.Z., Kooijman, P.G.C., Heszler, A.N., van der Meer, S., van Roon, R., Molleman, G.R.M., van den Muijsenbergh, M., Fransen, G.A.J., Bevelander, K.E. (Submitted). Citizens' perspectives on healthy weight approaches in low SEP neighborhoods: a qualitative study from a systems perspective.

Ter Bogt, M.J.J., Bevelander, K.E., Tholen, L., Molleman, G.R., van den Muijsenbergh, M, Fransen, G.A. (2023). Leverage point themes within Dutch municipalities’ healthy weight approaches: A qualitative study from a systems perspective. *PLOS ONE, 18*(6),e0287050. <https://doi.org/10.1371/journal.pone.0287050>
