# Supplementary material for: Mapping the dynamics of learning communities about Dutch healthy weight approaches: a causal loop diagram
Source: Arch Public Health. 2024 Dec 20;82:238. doi: 10.1186/s13690-024-01468-1 (PMC11660615; doi:10.1186/s13690-024-01468-1)
Supplement: Supplementary file 3 — Supplementary Material 3 [file 13690_2024_1468_MOESM3_ESM.docx]

**Additional file 3: Interview protocol 2022 [translated to English] – causal loop diagram about learning communities in five Dutch municipalities, 2022**

| This is the second part of the interview; the first part is described elsewhere (ter Bogt et al., 2023).  Meaning of colors and symbols   - Green: concepts to which the question refers (Gulikers et al., 2019; Wagemakers et al., 2010) - Purple: concept to which the question refers (van Mierlo et al., 2010) - Red: applies only to specific interviews (e.g., participants who were not invited for the first part of the interview, as described elsewhere (ter Bogt et al., 2023). - […]: name is filled in by researcher - Numbered questions: questions asked - : Possible in-depth question if answer was incomplete - a/b/c questions were always asked, unless the participant had already answered the question in a previous answer |
| --- |

*Introduction text – This was the first part of the conversation; we are now going to the second part. There have been two learning community meetings. We are now going to talk about the learning community meetings of the municipalities [municipality names].*

**Evaluation learning communities**

1. Think back to your participation in the learning community. What thoughts did you have? What did you like? What did you like less? *(General, Mutual willingness to reflect, Relation dimension, Task dimension, Suitability of the partners)*
2. What do you think of people involved in the learning community?
   1. When we talk about the learning community participants. What is going well? What things do you think are lacking? *(Prime mover, Suitability of the partners, Relation dimension, Trust)*
   2. Is there a driving force in the learning community? *(Prime mover)*
      *[If no]* How could this be designed? *[LC facilitator is not a member of the learning community]*

*[If not clearly stated in the answer]* How did you feel talking about the healthy weight approach?? *(Reflection, Mutual willingness to reflect)*

1. What have you told people around you about the learning community? (*Reflection, General, Suitablity of the partners, Task dimension, intrinsic motivation)*
   1. Why did/didn’t you tell people from your environment about the learning community?

**Learning community goals and roles**

*We are now going to talk about the goals of the learning community.*

1. What do you think the learning community is working toward? *(Task dimension)*
   1. Do you think the goal will be reached? Why/why not? *(Task dimension)*
2. What is your role within the learning community?? *(Involvement, Mutual willingness to reflect)*

*[Only for participants in part 2]* *The next question is about the healthy weight approach in [name municipality] that hinders or promotes healthy weight. These include for example activities and facilities aimed at nutrition, mental well-being, and poverty.* What do you want to achieve in this?

1. *You just mentioned your goal within the healthy weight approach. How does the learning community contribute to your previously stated goal for the healthy weight approach? (Goals – Action Scales Model)*

**Learning from monitoring**

What happens between the learning community meetings is observed. You and the other learning community participants reflect on this together during a learning community meeting. Then you make plans and carry out these actions. For example, you can map and adjust interim results in the learning community.

1. How do you experience this way of adjusting?
2. *[Not for only part 2 participants]* In your work, do you regularly reflect on how your activities and goals are progressing? How do you do that?
   1. How do you observe activities and goals in your work?
   2. How do you reflect on what you do in your work?
   3. How do you plan actions from this reflection?
   4. How do you carry out the planned actions?

*[Part 2 participants only]* Are there ways in which you were already involved in adjustments to of the healthy weight approach in [name municipality]? How do you do that?

**Individual learning**

1. What have you gained from the learning community so far?
   1. What do you learn from the learning community? *(Reflection, General, Learning)*

*[If not clearly stated in answer]* What knowledge did you gain? *(Identification)*

- 1. *[Not for only part 2 participants]* You just mentioned solutions for the points of attention that you indicated within the healthy weight approach. To what extent do you come to this solution through your participation in the learning community? Why/why not? (*Transformation*)
     1. [If not all solutions are covered] How is this different for [solution]?

*[Only part 2 participants]* During the learning community meeting, you mentioned various challenges and possible solutions within the healthy weight approach in [name municipality]. To what extent did you get this from the discussions in the learning community?

1. How do you benefit from the learning community contacts? *(Coordination, Reflection, Learning, Transformation)*

**Learning – interaction with wider network**

1. What do people around you who are not in the learning community think of the learning community and your participation?
   1. Do you involve them? *[If yes]* how do you involve them? *(Visibility, Reflection, Dimension, Coordination)*
2. How have you shared new knowledge from the learning community meetings with others outside the learning community? *(Coordination, Reflection, the learning effect occurs within their own network)*

*Introductory text – Now we have come to the final questions. These are about the actions that follow from the learning community meetings.*

**Acting**

1. How does the learning community influence what you do? *(Learning, Transformation, Acting, Change of routines)*
   1. How does what you do contribute to the solutions of the points of attention that you mentioned earlier within the healthy weight approach? (*Events*, *Transformation*, *Acting, Individual approach to institutional barriers)*
      1. [If not all solutions were covered] How is this different for [solution]?
   2. Have you started collaborating with other learning community participants through the learning community? What are you doing? *(Coordination, Reflection, Transformation, Relation dimension,, Acting)*
2. What results has the learning community already achieved? *(Transformation, Growth, Learning, Acting)*

*[If not fully answered]* What has the learning community achieved so far within the healthy weight approach?

1. Are there other important aspects of the learning community that we have not discussed so far?

*This was the final question. I would like to thank you for your time and participation in this conversation.***References**

Gulikers, J., Oonk, C. (2019). Towards a rubric for stimulating and evaluating sustainable learning. *Sustainability*, 11(4),969. <https://doi.org/10.3390/su11040969>

Ter Bogt, M.J.J., Bevelander, K.E., Tholen, L., Molleman, G.R., van den Muijsenbergh, M, Fransen, G.A. (2023). Leverage point themes within Dutch municipalities’ healthy weight approaches: A qualitative study from a systems perspective. *PLOS ONE, 18*(6),e0287050. <https://doi.org/10.1371/journal.pone.0287050>

Van Mierlo, B.C., Regeer, B., Amstel, M., Arkesteijn, M.C.M., Beekman, V., Bunders, J., et al. (2010). Reflexive Monitoring in action. A guide for monitoring system innovation projects. Communication and Innovation Studies, WUR; Athena Institute, VU

Wagemakers, A., Koelen, M.A., Lezwijn, J., Vaandrager, L. (2010). Coordinated action checklist: a tool for partnerships to facilitate and evaluate community health promotion. *Global Health Promotion*,17(3):17-28 . <https://doi.org/10.1177/1757975910375166>
