# Supplementary material for: Mapping the dynamics of learning communities about Dutch healthy weight approaches: a causal loop diagram
Source: Arch Public Health. 2024 Dec 20;82:238. doi: 10.1186/s13690-024-01468-1 (PMC11660615; doi:10.1186/s13690-024-01468-1)

**Additional file 4: The identified subthemes ordered and categorized under main themes per LC group – causal loop diagram about learning communities in five Dutch municipalities, 2022**

**Group A RQ1**

**
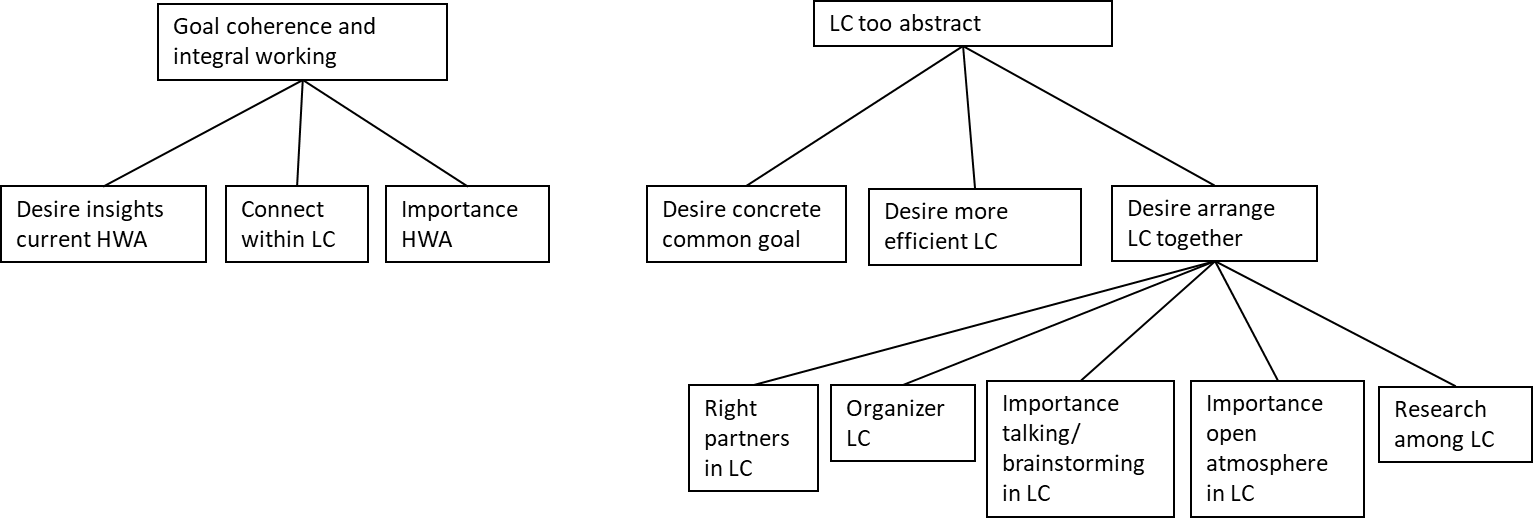
**

**
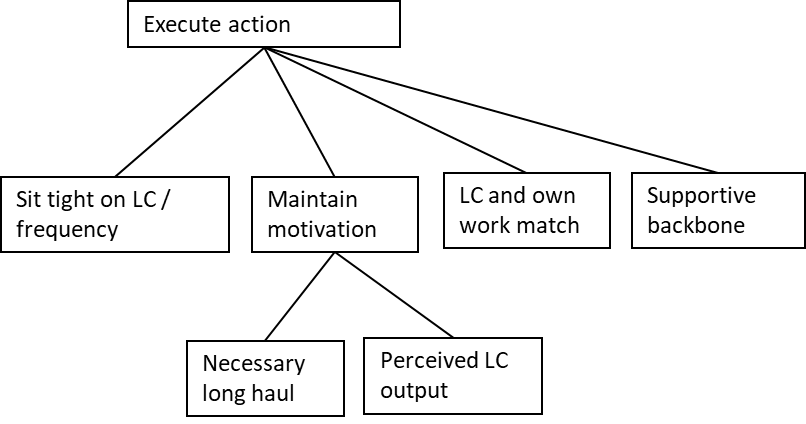
**

**Group A RQ2**

**
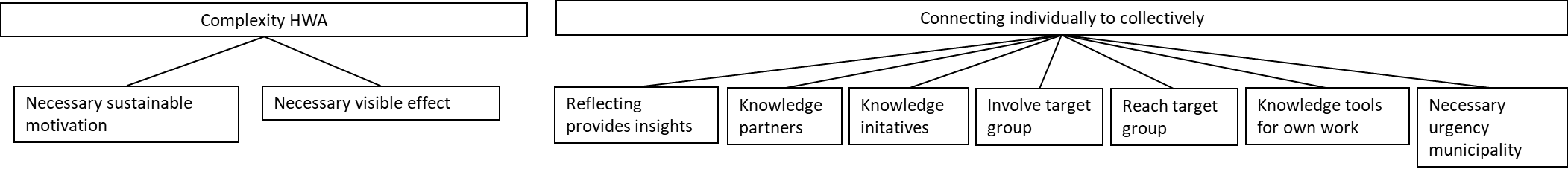
**

**Group A RQ3**


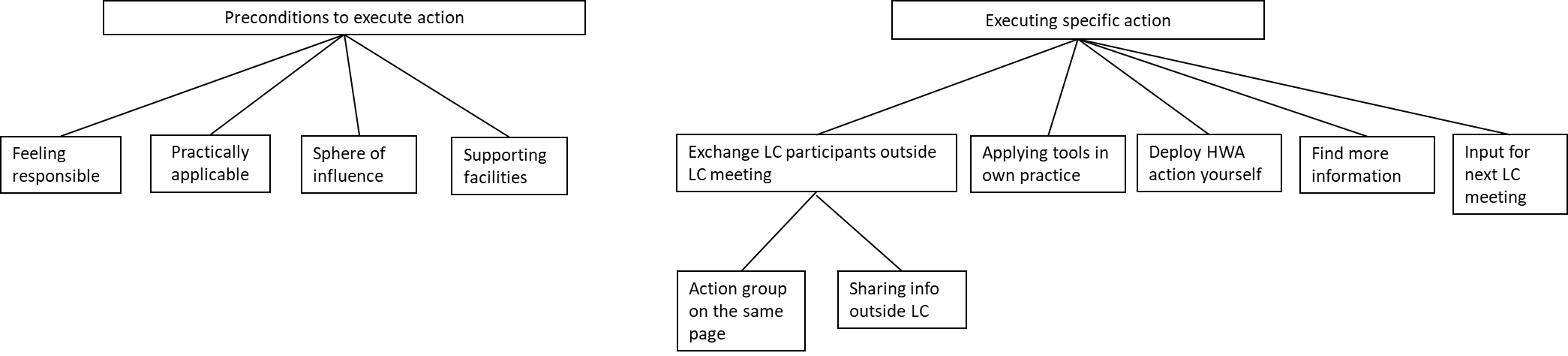


**Group B RQ1**

**
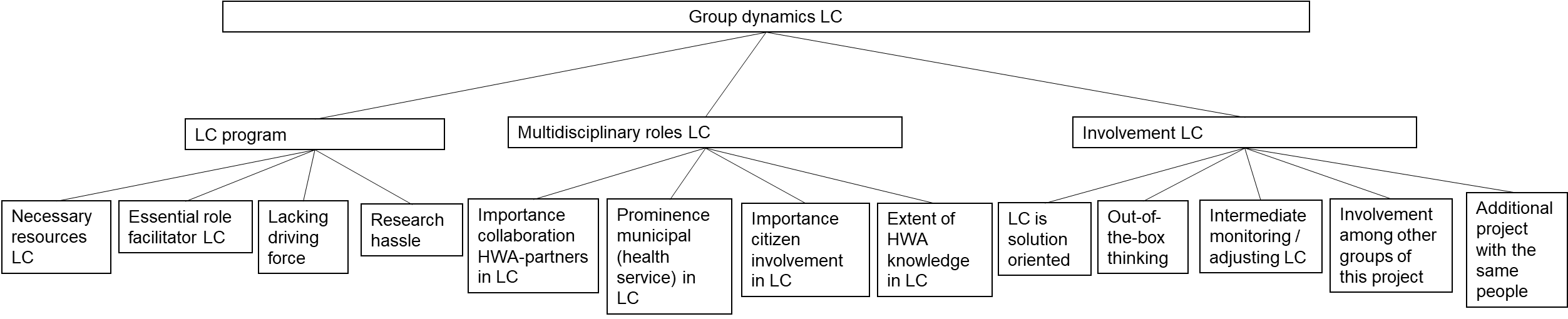
**


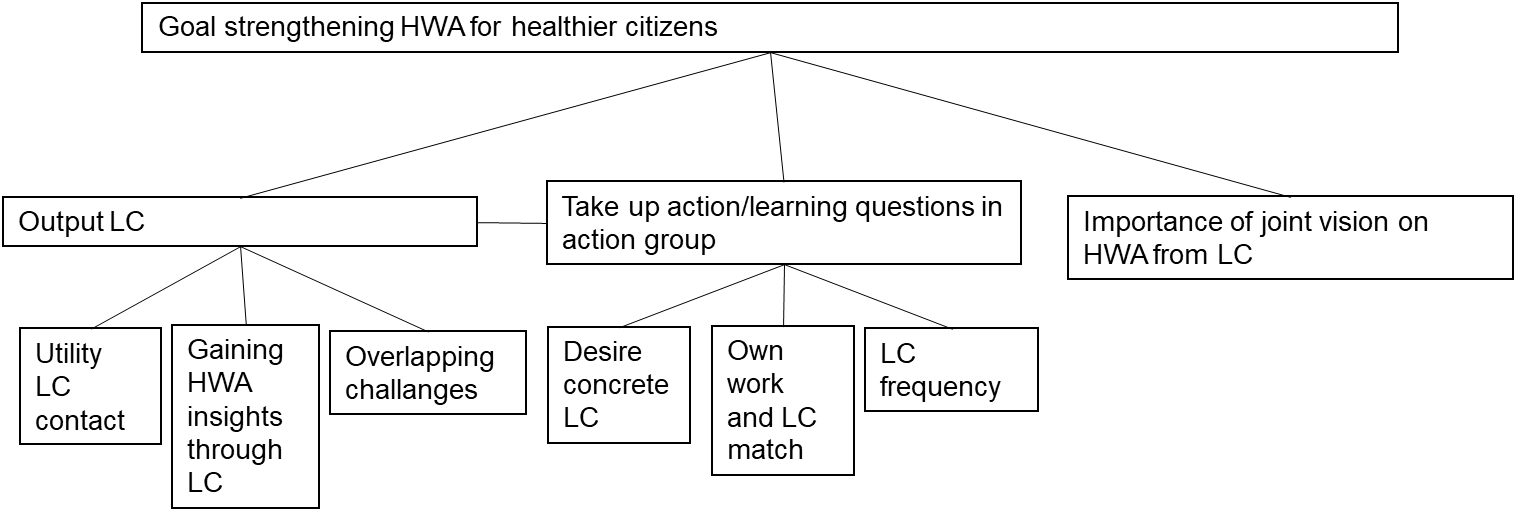


**Group B RQ2**


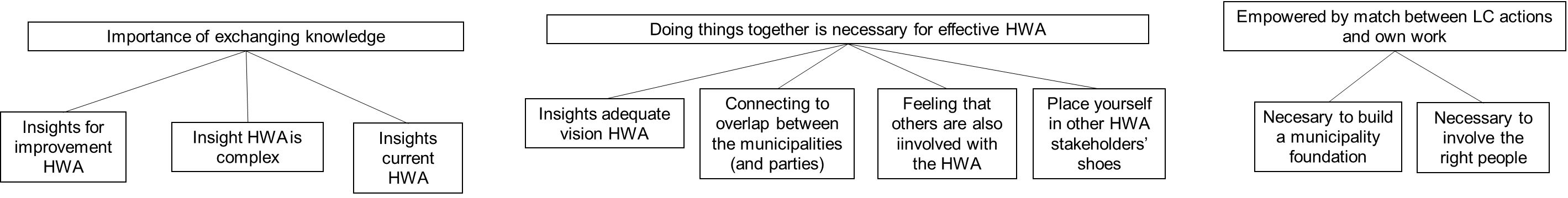


**Group B RQ3**


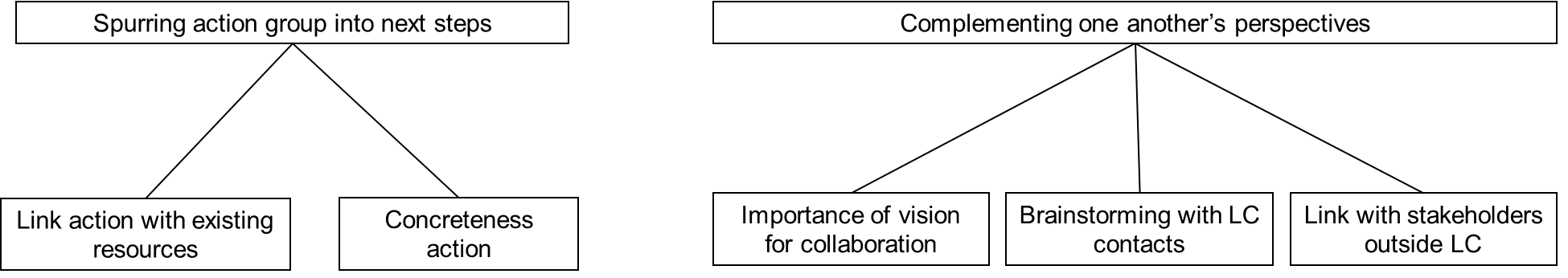

Supplement: Supplementary file 4 — Supplementary Material 4 [file 13690_2024_1468_MOESM4_ESM.docx]
