# Supplementary material for: Mapping the dynamics of learning communities about Dutch healthy weight approaches: a causal loop diagram
Source: Arch Public Health. 2024 Dec 20;82:238. doi: 10.1186/s13690-024-01468-1 (PMC11660615; doi:10.1186/s13690-024-01468-1)
Supplement: Supplementary file 5 — Supplementary Material 5 [file 13690_2024_1468_MOESM5_ESM.docx]

**Additional file 5: Dynamics within the CLD; feedback loops and described themes – causal loop diagram about learning communities in five Dutch municipalities, 2022**

| **Feedback loops^1^** | **Theme: Group dynamics in LC** |
| --- | --- |
| R1: 29 – 10 – 21 – 29 | Higher Jointly arranging the LC (13) was perceived to lead to more Appropriate partners (incl. citizens) involved in LC (9), which was perceived to lead to more Useful conversations with multidisciplinary LC participants (10). This was perceived to increase Feeling committed to a common HWA LC vision/goal (21) and Importance HWA involvement (5), which was perceived to increase Own motivation to execute LC actions (34). More Own motivation to execute LC actions (34) was perceived to increase Execute LC action (together) (e.g., brainstorming with members, applying tools in own practice) (4), which was perceived to increase Provide LC input (36). Subsequently, Jointly arranging the LC (13) was perceived to increase again. Higher Jointly arranging the LC (13) was also perceived to decrease Role research in LC (structured LC) (40). Lower Role research in LC (structured LC) (40), was perceived to decrease Guidance by facilitator (18) and subsequently was perceived to increase Jointly arranging the LC (13) again. Further, lower Guidance by facilitator (18) was perceived to decrease Efficient LC (19), which was perceived to increase LC content concrete (14). Higher LC content concrete (14) was perceived to lead to more Involvement LC participation (29), which was perceived to lead to more Useful conversations with multidisciplinary LC participants (10), and then was perceived to lead to more Feeling committed to a common HWA LC vision/goal (21). This was perceived to increase Involvement LC participation (29) again. More Useful conversations with multidisciplinary LC participants (10), also was perceived to lead to more Importance HWA involvement (5), which was perceived to increase Urgency to strengthen the HWA (41) and then was perceived to lead to more Feeling committed to a common HWA LC vision/goal (21) again. More Importance HWA involvement (5) and Feeling committed to a common HWA LC vision/goal (21) both lead to more Own motivation to execute LC actions (34), which was perceived to lead to more Provide LC input (36), and subsequently more Jointly arranging the LC (13) again. Increased Jointly arranging the LC (13) also was perceived to increase LC matches with members wishes (e.g., LC is solution oriented, adequate meeting duration) (15), which was perceived to lead to more Open atmosphere in LC (12), which also was perceived to increase Involvement LC participation (29). Higher Jointly arranging the LC (13) also was perceived to decrease Role research in LC (structured LC) (40), which was perceived to lead to higher Open atmosphere in LC (12), and then was perceived to increase Involvement LC participation (29) too. |
| R2: 13 – 40 – 18 – 13 |  |
| R3: 13 – 9 – 10 – 21 *or* 5 – 34 – 4 – 36 – 13 |  |
| R4: 13 – 40 – 12 – 29 – 10 – 5 *or* 21 – 34 – 4 – 36 – 13 |  |
| R5: 13 – 15 – 12 – 29 – 10 – 5 *or* 21 – 34 – 4 – 36 – 13 |  |
| R6: 13 – 40 – 18 – 19 – 14 – 29 – 10 - 5 or 21) – 34 – 4 – 36 – 13 |  |
| R7: 41 – 21 – 29 – 10 – 5 – 41 |  |
| **Feedback loops** | **Theme: Gaining insights through exchange in LC** |
| R8: 29 – 10 – 22 – 27 - 16 – 29 | Higher Involvement LC participation (29) was perceived to lead to more Useful conversations with multidisciplinary LC participants (10), which was perceived to increase Connecting with useful LC contacts (22). This was perceived to lead to Better understanding overlap between municipalities and partners (e.g., HWA challenges) (27), which was perceived to lead to more formulated (individual or shared) LC actions (16), and then to higher Involvement LC participation (29) again. More Useful conversations with multidisciplinary LC participants (10) also was perceived to lead to more Reflecting by putting yourself in another HWA stakeholder’s shoes (24), which was perceived to increase Better understanding HWA complexity (e.g., HWA initiatives, HWA points of improvement) (26) and then was perceived to increase Better understanding overlap between municipalities and partners (e.g., HWA challenges) (27). More Better understanding overlap between municipalities and partners (e.g., HWA challenges) (27) was perceived to increase LC was perceived to lead to formulated (individual or shared) LC actions (16). More LC was perceived to lead to formulated (individual or shared) LC actions (16) was perceived to lead to more Involvement LC participation (29) again, and then was perceived to lead to more Useful conversations with multidisciplinary LC participants (10) again. |
| R9: 10 – 24 –26 – 27 – 16 – 29 – 10 |  |
| **Feedback loops** | **Theme: Conditions to execute LC actions** |
| R10: 34 – 4 – 3 –– 34 | Higher Own motivation to execute LC actions (34) was perceived to increase Execute LC action (together) (e.g., brainstorming with members, applying tools in own practice) (4), which was perceived to increase Perceived output (visible effects) (3). This was perceived to lead to more Own motivation to execute LC actions (34) again. Higher Execute LC action (together) (4) also was perceived to increase Effectiveness HWA (43), which was perceived to decrease Overweight prevalence in municipality (42). This was perceived to lead to decreased Importance HWA involvement (5), which was perceived to decrease Own motivation to execute LC actions (34) and then was perceived to decrease Execute LC action (together) (4). Higher Execute LC action (together) (e.g., brainstorming with members, applying tools in own practice) (4) also was perceived to lead to more Provide LC input (36), and then to more Jointly arranging the LC (13). This was perceived to increase Match LC and own work (31), which was perceived to increase Feeling responsible for LC actions (33) and LC content concrete (14). More LC content concrete (14) was perceived to lead to more Involvement LC participation (29), which was perceived to increase Useful conversations with multidisciplinary LC participants (10) and then was perceived to increase Importance HWA involvement (5). Higher Feeling responsible for LC actions (33) and Importance HWA involvement (5) were both perceived to increase Own motivation to execute LC actions (34) again. |
| B1: 4 - 43 – 42 - 5 – 34 – 4 |  |
| R11: 34 – 4 – 36 – 13 – 31 – 33 (*or* 14 – 29 – 10 – 5) – 34 |  |
| **Feedback loops** | **Interconnected themes** |
| R12: 4 – 36 – 13 – 9 – 23 – 26 – 38 – 4 | Higher Execute LC action (together) (e.g., brainstorming with members, applying tools in own practice) (4) was perceived to increase Provide LC input (36), which was perceived to increase Jointly arranging the LC (13). This was perceived to lead to more Appropriate partners (incl. citizens) involved in LC (9), which was perceived to lead to more Extent of HWA knowledge in LC (23) and then to more Better understanding HWA complexity (e.g., HWA initiatives, HWA points of improvement) (26). This was perceived to increase Involving partners outside LC during execution (38) and then was perceived to increase Execute LC action (together) (e.g., brainstorming with members, applying tools in own practice) (4) again. More Appropriate partners (incl. citizens) involved in LC (9) also was perceived to lead to more Useful conversations with multidisciplinary LC participants (10), which was perceived to lead to more Connecting with useful LC contacts (22). This was perceived to lead to Better understanding overlap between municipalities and partners (e.g., HWA challenges), which was perceived to lead to more formulated (individual or shared) LC actions (16), and then was perceived to increase Execute LC action (together) (e.g., brainstorming with members, applying tools in own practice) (4) again. |
| R13: 4 – 36 – 13 – 9 – 10 – 22 – 27 – 16 – 4 |  |

^1^All most significant reinforcing (R) and balancing (B) feedback loops are shown in the Table. As some feedback loops are connected throughout, some feedback loops can be combined into one larger feedback loop, as shown in the Figure. These combined feedback loops are not explicitly mentioned in the Table.
